# A Computer-Based Methodology to Design Non-Standard Peptides Potentially Able to Prevent HOX-PBX1-Associated Cancer Diseases

**DOI:** 10.3390/ijms22115670

**Published:** 2021-05-26

**Authors:** Maria Rita Gulotta, Giada De Simone, Justin John, Ugo Perricone, Andrea Brancale

**Affiliations:** 1 Molecular Informatics Unit, Fondazione Ri.MED, Via Filippo Marini 14, 90128 Palermo, Italy; gdesimone@fondazionerimed.com (G.D.S.); uperricone@fondazionerimed.com (U.P.); 2 NRN Tech LTD, Henstaff Court, Llantrisant Road, Groesfaen CF72 8NG, UK; justin@nrntech.co.uk; 3 School of Pharmacy and Pharmaceutical Sciences, Cardiff University, King Edward VII Avenue, Cardiff CF10 3NB, UK; brancalea@cardiff.ac.uk

**Keywords:** HOX, PBX, Protein-Protein Interactions, Residue Scanning, Molecular Dynamics, MM-GBSA, Cancer, Non-standard amino acids

## Abstract

In the last decades, HOX proteins have been extensively studied due to their pivotal role in transcriptional events. HOX proteins execute their activity by exploiting a cooperative binding to PBX proteins and DNA. Therefore, an increase or decrease in HOX activity has been associated with both solid and haematological cancer diseases. Thus, inhibiting HOX-PBX interaction represents a potential strategy to prevent these malignancies, as demonstrated by the patented peptide HTL001 that is being studied in clinical trials. In this work, a computational study is described to identify novel potential peptides designed by employing a database of non-natural amino acids. For this purpose, residue scanning of the HOX minimal active sequence was performed to select the mutations to be further processed. According to these results, the peptides were point-mutated and used for Molecular Dynamics (MD) simulations in complex with PBX1 protein and DNA to evaluate complex binding stability. MM-GBSA calculations of the resulting MD trajectories were exploited to guide the selection of the most promising mutations that were exploited to generate twelve combinatorial peptides. Finally, the latter peptides in complex with PBX1 protein and DNA were exploited to run MD simulations and the ΔG_binding_ average values of the complexes were calculated. Thus, the analysis of the results highlighted eleven combinatorial peptides that will be considered for further assays.

## 1. Introduction

Human phenotype development, evolution and physiopathological processes are regulated by several key actors. Among these, *HOX* genes have been associated with the control of the final morphology [1,2]. Their increase or decrease in activity can often result in homeotic transformations provoking the formation of structures or organs in erroneous locations within the organism. Three different levels of *HOX* genes evolutionary conservation have been identified: (1) at a molecular level, they all encode homeodomain transcription factors [3]; (2) at a structural level, *HOX* genes are usually organised in complexes, reflecting their phylogeny and regulatory aspects of their expression [4,5], and (3) at a functional level, they trigger similar effects in most animals and can work in substitution of an orthologue in other species [6].

When becoming highly dysregulated and overexpressed, *HOX* genes have been associated with a wide range of both solid and haematological cancers [7].

For this reason, the processes modulated by *HOX* genes have been extensively studied providing a substantial, although not exhaustive, analysis of them. Recent studies highlighted that *HOX* genes also contribute to organogenesis [8] by influencing a huge number of cellular functions such as differentiation, proliferation, migration or death [9]. 

**Figure 1:**
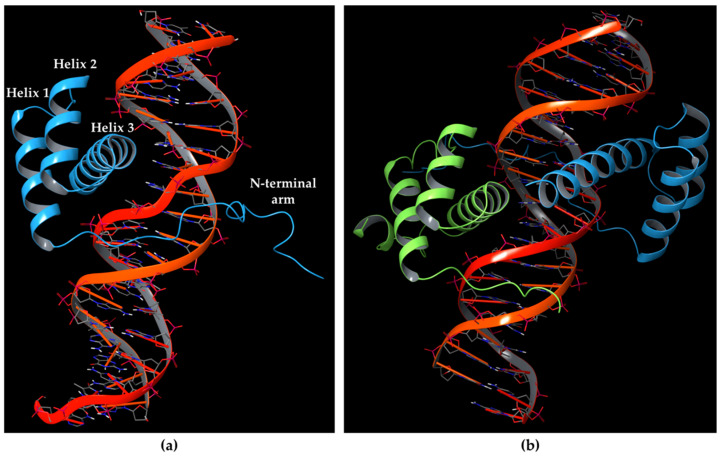
(**a**) Homeodomain of HOXA9 protein (light blue chain) showing a triple-helix structure where helix 3 insert the DNA major groove, while the N-terminal arm establishes contacts with DNA minor groove (PDB ID: 1PUF [27]); (**b**) HOXA9-PBX1 cooperative binding in presence of DNA retrieved from PDB 1PUF [27], where the red double-helix structure is DNA bound to the Homeobox protein HOXA9 (light blue chain) and Pre-B-cell leukaemia transcription factor-1 PBX1 (green chain).

HOX proteins consist of two highly conserved portions: the hexapeptide (HX) motif and the homeodomain (HD). The HX motif establishes interactions with protein members of the PBC class, such as Pre-B-cell Leukemia Homeobox (PBX) proteins in humans [10]; while the HD motif corresponds to the DNA-binding domain. The HD folds into a triple-helix structure, including the N-terminal arm binding the minor groove of DNA, and helix 3 (also named the recognition helix) contacting the DNA in the major groove, as depicted in Figure 1a. Residues involved in HOX HD helices 1 and 3 have shown to be the most conserved, and other amino acids of the N-terminal arm and loops between the helices have been reported well conserved among HOX proteins. Furthermore, the conservation of HD sequences is highly shared between HOX proteins, raising the issue of how they employ functional specificity [11,12,13].

Indeed, the homeodomain of HOX proteins does not exhibit high specificity for DNA, by taking part in the molecular recognition through five amino acids [14]. In this context, functional studies in the field of cancer and developmental biology highlighted the role of PBX proteins as HOX co-factors [15,16,17], whereas PBX family members bind to HOX proteins 1-11 [18,19,20]. These proteins may establish a cooperative binding to DNA [21,22] as depicted in Figure 1b, indicating that the interaction of HOX proteins to PBX influences the DNA-binding of HOX by fostering a greater specificity [23]. Furthermore, the HOX co-factors play other key roles influencing transcriptional events, by recruiting the RNA polymerase II and III or transcriptional inhibitors like HDAC, and post-translational events, by fostering the entry of HOX proteins into the nucleus.

Four different types of *PBX* genes (PBX1-4) are encoded in the human genome. As for HOX proteins, *PBX* genes also encode evolutionarily conserved homeodomains and other highly conserved regions [16]. PBX proteins present two nuclear localization signals (NLSs) in the homeodomain and a nuclear export sequence (NES) [24,25,26].

PBX proteins may participate in a DNA binding consensus through the formation of strong complexes with HOX1-11 proteins [21,28,29]. The involved interactions have been shown to exhibit a highly conserved interaction mode between the HX motif of HOX and the three amino acid loop extension (TALE) or three-amino acid insertion peptide of PBX, which is located between helices 1 and 2 of the homeodomain [18,27,29,30,31,32,33].

In 1995, Knoepfler and Kamps [14] identified the minimal sequences that enable HOXB8 and HOXA5 proteins to bind cooperatively PBX1 protein, by performing deletion mutagenesis on the above-mentioned HOX proteins. This minimal sequence was the conserved pentapeptide motif *Y/F-P-W-M-R/K*. In particular, mutations at tryptophan residue did not produce binding abrogation. Mutations of Trp135 to phenylalanine (W135F) or alanine (W135A) on HOXB8 did not alter the DNA binding but completely abolished the cooperativity of HOXB8 with PBX1. Met136 was also shown to be important but not essential for the DNA binding of PBX1, whereas its mutation to isoleucine (M136I) or alanine (M136A) strongly disrupted the cooperativity of HOXB8 with PBX1. Therefore, both residues, Trp135 and Met136, were considered crucial for the protein/DNA interaction, with particular attention for Trp135 [32], since it was the only conserved amino acid among all HOX proteins pentapeptide sequences. Knoepfler and Kamps also assumed that the pentapeptide HOX motif stabilizes the trimeric HOX-PBX1-DNA complex by bringing a portion of the HOX protein surface into contact with PBX1 and enhancing DNA binding in the presence of PBX1 [14]. However, the X-ray crystallographic structures of HOXB1-PBX1 and HOXA9-PBX1 in the presence of DNA revealed that the protein-protein-DNA contacts are stabilized by the interaction between HOX and PBX mediated not by a simple pentapeptide sequence, but by a conserved hexapeptide sequence in HOX proteins [3,18,27,31].

In 1999 Piper et al. [18] found that a minimal portion of HOX containing hexapeptide and homeodomain was able to cooperatively stabilize DNA binding with PBX1. Hence, the identified consensus hexapeptide motif from HOX proteins was *φ*-Y/F-P-W-M-K/R, where *φ* stands for a hydrophobic residue. As reported above, tryptophan and methionine were conserved.

X-ray crystallographic structures of the ternary complex, HOX-PBX1-DNA (e.g., HOXA9 in PDB 1PUF, Resolution: 1.90 Å; and HOXB1 in PDB 1B72, Resolution: 2.35 Å) [27] revealed that HOX protein and PBX1 establish contacts with opposite DNA faces, burying 2400 Å^2^ of protein and DNA surface. The HOX hexapeptide mediates contacts with PBX1 within a hydrophobic pocket located between the TALE region and helix 3 of the PBX1 homeodomain.

PCR site-selection experiments performed by Piper et al. [18] allowed us to identify the optimal HOXB1-PBX1 binding site on the 20 bp duplex DNA oligonucleotide, i.e., 5′-ATGATTGATCG-3′ [34].

**Figure 2:**
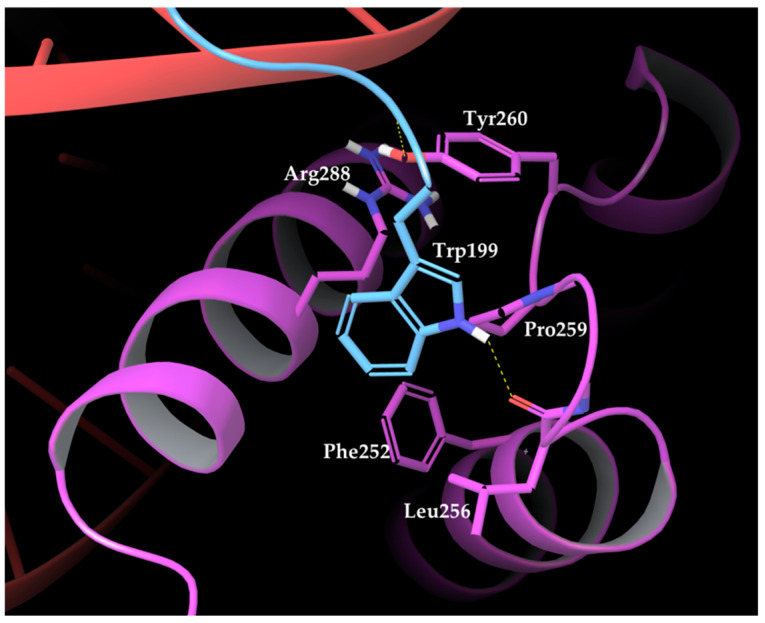
Amino acids composing PBX1 binding pocket (purple residues and chain) surrounding HOXA9 Trp199 (light blue chain) [27].

The PDB structure resolved by La Ronde-Le Blanc and Wolberger [27] revealed that the interactions between HOXA9 and PBX1 are mediated by HOXA9 hexapeptide, consisting of residues 196 to 201 with the AANWLH sequence linked to the PBX1 homeodomain. As observable in the PBD structures, the hexapeptide residues mediate mainly hydrophobic contacts, whereas HOXA9 Trp199 side-chain inserts into a hydrophobic pocket of PBX1 formed by the C terminus of helix 3, a handle between helices 3 and 4, and the three–amino acid insertion. The key interactions involve HOXA9 Trp199 with its indole ring that forms van der Waals contacts with several PBX1 residues, such as the Phe252 side chain in helix 1, Leu256 within the TALE region, Pro259 and Tyr260 following the TALE peptide, and Arg288 in helix 3. Furthermore, Trp199 is highly buried into the PBX1 binding pocket by forming a hydrogen bond between the indole nitrogen and the backbone carbonyl of PBX1 Leu256 (Figure 2).

The nitrogen atom at HOXA9 Leu200 backbone establishes van der Waals contacts with Ly292 and a hydrogen bond with Tyr260 hydroxyl group within the binding pocket of PBX1. Finally, His201 of HOXA9 hexapeptide forms a hydrogen bond with Lys292 of PBX1.

Moreover, Piper et al. [18] on PBX1 conducted some mutational studies focused on the hexapeptide-contacting residues Leu252 and Pro259, which were substituted for alanine, triggering the disruption of the interactions with the hexapeptide in vitro and in a yeast two-hybrid assay [35]. Furthermore, deletion assays at the three–amino acid insertion abolished the cooperative binding of PBX1 with HOX proteins [36]. On the other hand, the deletion of the HOX hexapeptide caused the disappearance of cooperative interactions between PBX1 and HOX proteins [14,29,37].

Although 3D structures of the ternary complex HOX-PBX-DNA have been experimentally solved, the drug discovery process for this protein-protein interaction (PPI) has met some issues typical of targeting a PPI through designing potential effective small molecule inhibitors [38,39]. However, an accepted strategy is to target the HOX-PBX binding interface at the highly conserved residues involving HOX hexapeptide and exploiting the hydrophobic nature of the PBX protein binding pocket. In the last decades, a small molecule inhibitor of this interaction was identified. However, its K_D_ was in the micromolar range (65 µM) and it was neglected for further experimental assays or clinical trials [40]. On the other hand, in the last years, several peptides have been designed based on the hexapeptide consensus motif of HOX proteins, to work as a competitive antagonist of HOX-PBX binding [41]. The most promising peptide among these was HXR9, an 18-amino acid peptide containing the hexapeptide sequence together with a polyarginine portion.

HXR9 was first shown to be cytotoxic to melanoma cell lines and primary melanoma cells and registered a reduction of B16F10 murine melanoma tumours growth in an orthotropic model [42]. Other experimental studies reported that HXR9 was able to inhibit the growth of several tumour types in mouse xenograft models, including non-small cell lung [43], breast [44], ovarian [44], and prostate cancer [45], and mesothelioma [46], melanoma [47], and meningioma [48]. 

Recently, modifications performed on the HXR9 sequence shed light on another peptide, i.e., the HTL001 peptide [49] with the sequence WYPWMKKHHRRRRRRRRR, that was tested in cancer cells representative of 14 human and animal malignancies. HTL001 registered selective toxicity for cancer cells and safety for normal cells. To date, this peptide has reached the human clinical trials that are ongoing to assay the efficacy and safety. However, the mechanism associated with HOX-PBX inhibition and the resulting cell death through employing the HTL001 peptide is still to be fully elucidated, although generally in most solid tumours cell death is mediated by apoptosis [42,44,45,46,50].

**Figure 3:**
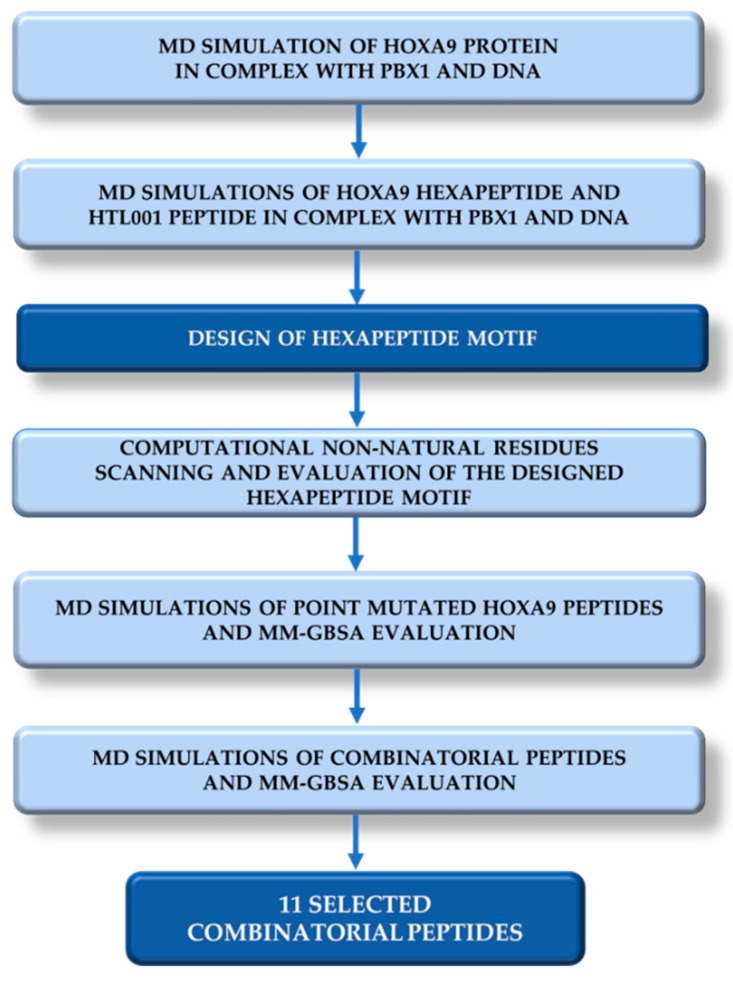
Overview of the computational workflow performed to identify the eleven combinatorial peptides potentially inhibiting HOX-PBX1 cooperative binding.

In this article, we describe a computer-based strategy to design novel peptides including non-standard amino acids to potentially bind PBX1 and inhibit HOX-PBX1 interaction (Figure 3). For this purpose, a Molecular Dynamics (MD) simulation of 200 ns was performed on the ternary complex HOXA9-PBX1-DNA retrieved from the Protein Data Bank [51] to identify key residues and interactions [52]. Two other MD were run, one on HOXA9 hexapeptide (196-AANWLH-201) from PDB 1PUF and the other on the patented core peptide HTL001 without polyarginine coil in complex with PBX1-DNA. The resulting MD trajectories were then used to compute MM-GBSA (Molecular Mechanics–Generalised Born Surface Area) calculations to obtain ΔG_binding_ average values as references for the design of the new peptides. Subsequently, the HOXA9 hexapeptide sequence was submitted to a point mutational scanning exploiting a non-natural amino acid database populated by the Swiss Institute of Bioinformatics [53,54]. The mutants were selected according to ΔΔG_affinity_ and ΔΔG_stability_ values and, in complex with PBX1-DNA, were further explored by MD simulations and MM-GBSA calculations. All those complexes point-mutated peptide-PBX1-DNA reporting ΔG_binding_ average values lower compared to the reference ΔG_binding_ average values (involving HOXA9 hexapeptide and HTL001 core peptide) were chosen for the next steps of the work. Thus, the most promising mutated peptides were used as starting point to generate twelve combinatorial peptides. The newly generated peptides were reprocessed in MD and MM-GBSA calculations as peptide-PBX1-DNA complex. Finally, eleven peptides showed promising ΔG_binding_ values compared to the HOXA9 hexapeptide and HTL001 peptide.

## 2. Results and Discussion

### 2.1. Molecular Dynamics Simulation of HOXA9-PBX1-DNA Complex

The first step of this work was the analysis and selection of a high-quality PDB structure of the trimeric complex HOXA9-PBX1-DNA currently available in the Protein Data Bank [51] to perform an MD simulation. For this purpose, PDB 1PUF (resolution 1.90 Å) [27] was chosen for a 200 ns MD simulation, to analyse and identify the most stable interactions and key residues for both proteins. The complex stability was also investigated by reporting the RMSD plot illustrated in Figure S1 in Supplementary materials, which showed a stable behaviour of the system.

The trajectory was further analysed to retrieve the most frequent interactions established between the HOX and PBX proteins. MD frames were clustered in 10 groups based on the RMSD matrix by using both protein backbone and sidechains and setting a frequency of 10 steps at which the frames were analysed. The centroid frames for the most abundant clusters were: frame 880 (representative for 63 frames), frame 70 (representative for 34 frames), frame 540 (representative for 28 frames), frame 360 (representative for 22 frames), and frame 270 (representative for 15 frames) [55].

The observed interactions in these frames were analysed and considered as the most stable and frequent during the trajectory. Table 1 lists the key residues involved in contacts between the two proteins, whereas for HOXA9 only residues involved in the PBX-contacting hexapeptide region 196-AANWLH-201 were included in Table 1.

As it can be observed, Trp199 was detected as the key residue showing the majority of the interactions with PBX1 residues. This result was in agreement with information reported in the literature highlighting this tryptophan amino acid [14] as the fundamental one and was considered for the design of novel peptides described in the next sections.

### 2.2. MD Simulations of HOXA9 Hexapeptide and HTL001 Core Peptide in Complex with PBX1 Protein

**Figure 4:**
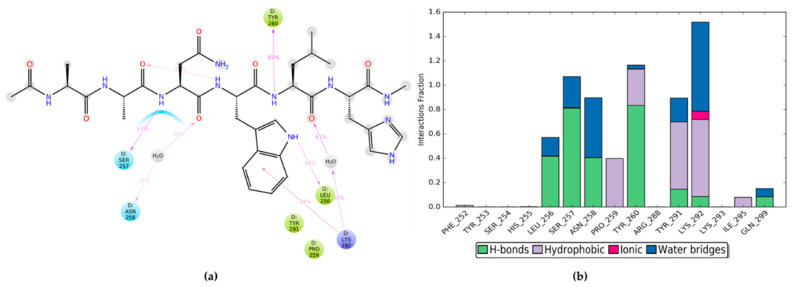
(**a**) Diagram of HOXA9 hexapeptide interactions with PBX1 residues during MD simulation; (**b**) bar chart of protein-ligand interaction occurrences during MD simulation.

Before proceeding with the design of novel peptides, other investigations were considered necessary to explore more in details the binding mode of PBX1 with the minimal active HOXA9 sequence (hexapeptide). For this reason, an MD simulation of 200 ns was performed by using the PDB 1PUF including HOXA9, PBX1 and DNA, where HOX protein was modified by deleting all those amino acids not included into the PBX-contacting hexapeptide 196-AANWLH-201. The protein and ligand RMSD plot of the trajectory was analysed by reporting the trend illustrated in Figure S2 in Supplementary materials. The HOXA9-PBX1 interaction diagram and bar chart for this MD simulation are depicted in Figure 4.

**Figure 5:**
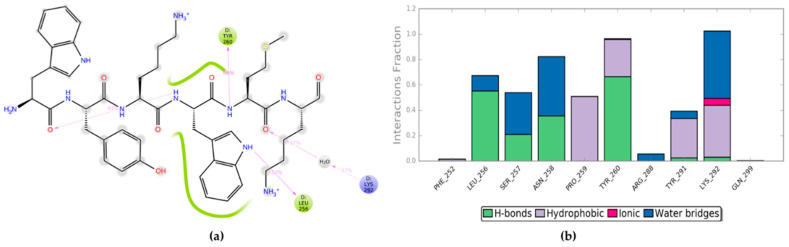
(**a**) Diagram of HTL001 hexapeptide (WYKWMK) interactions with PBX1 residues during MD simulation; (**b**) bar chart of PBX1-HTL00 hexapeptide interaction occurrences during MD simulation.

As mentioned above, to date the patented peptide HTL001 is being clinically employed, showing promising results by preventing HOX-PBX cooperative binding [49]. In detail, the HTL001 peptide sequence incorporates the hexapeptide WYKWMK responsible for the binding affinity with PBX proteins and a polyarginine coil that functions as a cell-penetrating fragment. Therefore, PDB 1PUF was used for another MD simulation of 200 ns, where HOXA9 hexapeptide was modified and energy minimised to reproduce the HTL001 hexapeptide sequence (WYKWMK), without the polyarginine segment, in complex with PBX1 and DNA. Then, the entire trajectory was monitored and the RMSD plot of PBX1 protein and HTL001 hexapeptide is depicted in Figure S3 in Supplementary materials. Finally, the ligand interaction diagram and the bar chart of protein-ligand interaction occurrences are shown in Figure 5.

The two above-described MD trajectories were processed to compute MM-GBSA calculations, to analyse the ΔG_binding_ average values, that are reported in Table 2. These results were used as references for the next steps of this work to compare MM-GBSA outputs of the designed peptides below described in complex with PBX1.

### 2.3. Residue Scanning of Point-mutated Peptides and Related MD Simulations and MM-GBSA Calculations

HOX proteins have been extensively studied, thus experimental evidence [14,18] highlighted the consensus HOX hexapeptide sequence φ-Y/F-P-W-M-R/K (where φ is a hydrophobic residue) [18]. This consensus motif has been shown responsible for the cooperative binding to PBX proteins and to increase specificity for DNA. Therefore, based on this information from the literature and considering the above-described computational data that shed light on Trp199 as a key amino acid for the interactions, a peptide motif was designed to guide the next steps of this work, as follows:

X_1_-X_2_-X_3_-W-X_4_-X_5_

The amino acid tryptophan was maintained from the consensus HOX motif and the other positions, X_1_ to X_5_ amino acids, were substituted with non-standard (or non-natural) residues, to generate peptides with different sequence from HTL001.

For this purpose, the SwissSidechain database of non-natural residues populated by the Swiss Institute of Bioinformatics [53] was used. SwissSidechain is a structural and molecular mechanics database of 200 amino acid with non-standard side chains (both D and L conformations), that was developed to study in silico their insertion into natural peptides or proteins. In this work, HOXA9 hexapeptide retrieved from PDB 1PUF was used as a reference to design and identify peptides incorporating non-standard amino acids. The aim was to find peptides able to increase the HOX-PBX inhibitory activity of HTL001 peptide. Indeed, Gfeller et al. [54] demonstrated very good reliability of the 

SwissSidechain database based on a comparison between predicted and experimental binding free energies for a BCL9 peptide targeting beta-catenin. These results indicated that such non-natural residues can be used to design novel protein-protein inhibitors. The non-standard side chains of this database were designed based on structural information collected from the Protein Data Bank (PDB) [51] and also commercially available non-natural amino acids. To avoid potential perturbations to peptides or proteins conformation, all those residues that might induce modifications of the backbone (such as β-homo, cyclic or aromatic backbones, or proline derivatives) were not included in the database. Therefore, a total of 200 non-natural side chains populated the SwissSidechain database, where 141 residues were extracted from the Protein Data Bank [51]. All these non-standard residues were collected and used in this work. This database was merged with the non-natural residue library already available in the Schrödinger suite, achieving overall 220 non-standard amino acids. Then point mutations were performed on the HOXA9-PBX1-DNA complex (PDB 1PUF [27]) by running the residue scanning for each of the five X_1-5_ amino acid positions present in the designed peptide motif and corresponding to Ala196, Ala197, Asn198, Leu200 and His201 of HOXA9 protein. Only Trp199 was maintained unchanged due to its relevance for PBX1 binding [32].

After running the residue scanning calculations, the first four most promising non-standard amino acids were selected for each X_1-5_ residue of the designed peptide. For this purpose, the residues were chosen according to the following three criteria:

(1) A cut-off of −3.0 kcal/mol for the affinity free-energy difference (ΔΔG_affinity_) between mutated and wild-type complexes was tuned according to a study performed by Beard et al. [56]. The authors demonstrated a scale factor of 3 to relate the predicted ΔΔG_affinity_ values of a mutation through the Schrödinger suite and the experimental energies. According to their study, a computed cutoff of −3 kcal/mol was applied for ΔΔG_affinity_ between the mutant and the wild-type form of the protein to predict the key pointed mutations [57];

(2) Negative values for stability free-energy difference (ΔΔG_stability_) between mutated and wild-type complexes were considered affordable. Due to the lack of a defined tertiary structure of HOX hexapeptide, it was assumed that mutations should not significantly affect the ΔΔG_stability_ of the peptide. Therefore, the ΔΔG_stability_ values were considered acceptable if negative;

(3) Commercial availability of non-standard amino acids.

In Table 3 the selected non-standard amino acids are listed, whereas X_1_, X_2_, X_3_, X_4_ and X_5_ amino acids provided respectively 119, 10, 52, 35 and 5 acceptable mutations. However, only the best four non-natural amino acids were chosen to proceed with the studies according to the three above-listed criteria.

Each point-mutated HOXA9 peptide was further processed to run an MD simulation of 200 ns in complex with PBX1-DNA to explore the binding stability of each complex for a total of 20 MD simulations. The analysis of the MD outputs showed that the peptides mainly established the crucial interactions with Trp199. The MD trajectories were further processed to compute MM-GBSA calculations, to compare the resulting ΔG_binding_ average values of the complexes to the ΔG_binding_ of the wild-type system, whereas the complex including HOXA9 hexapeptide reported ΔG_binding-HOXA9_ = –58.1922 kcal/mol and the one incorporating HTL001 core peptide showed ΔG_binding-HTL001_ = –53.6882 kcal/mol. All those mutations reporting ΔG_binding_ average values lower than the two above-mentioned ones were considered for the next steps of the work. In Table 3 MM-GBSA values are reported for each point-mutated peptide.

In detail, for position X_1_ of the designed hexapeptide motif three non-natural amino acids (CIR, MTR and ALC) showed low ΔG_binding_ average values, for position X_2_ only residue TBP reported good ΔG_binding_ average value; for position X_3_ only the second non-standard amino acid 0BN was considered for further analysis; for X_4_ all the four amino acids (PBF, CP3, QU4 and ANT) showed good ΔG_binding_ average values; and finally, for X_5_ none of the four non-natural amino acids was suitable to be used for the next steps (see Table 4 for MM-GBSA values).

### 2.4. Combinatorial Peptides Generation and Related MD Simulations and MM-GBSA Calculations

The above-described MM-GBSA calculations allowed to select overall nine mutations that were combined based on the designed peptide motif (X_1_-X_2_-X_3_-W-X_4_-X_5_), by employing HOXA9 hexapeptide scaffold. Thus, twelve combinatorial peptides were generated, as listed below, where only two amino acids of HOXA9 were maintained: tryptophan due to its crucial role in PBX-binding and histidine because mutations did not report good ΔG_binding_ average values.

1 CIR–TBP–0BN–Trp–PBF–His
2 CIR–TBP–0BN–Trp-CP3–His
3 CIR–TBP–0BN–Trp-QU4–His
4 CIR–TBP–0BN–Trp–ANT–His
5 ALC–TBP–0BN–Trp–PBF–His
6 ALC–TBP–0BN–Trp–CP3–His
7 ALC–TBP–0BN–Trp–QU4–His
8 ALC–TBP–0BN–Trp–ANT–His
9 MTR–TBP–0BN–Trp–ANT–His
10 MTR–TBP–0BN–Trp–CP3–His
11 MTR–TBP–0BN–Trp–QU4–His
12 MTR–TBP–0BN–Trp–ANT–His

These combinatorial peptides in complex with PBX1 protein and DNA were used to run MD simulations of 200 ns per complex. Thus, twelve MD were run and the RMSD values were plotted for each ternary complex DNA-protein-peptide, as depicted in Table S1 in Supplementary materials. On the other hand, Table S2 in Supplementary materials depicts the bar charts of protein-ligand interactions and the plots illustrating the frequency of the interactions during the MD trajectories. The analysis of these RMSD plots confirmed the stability of the simulated systems. Furthermore, the bar charts and the interaction frequency plots highlighted that the twelve combinatorial peptides met most of the key interactions previously identified from the analysis of PDB 1PUF structure and MD simulations of HOXA9 protein, HOXA9 hexapeptide and HTL001 peptide with PBX1 and DNA. More details about statistics of H-bonds and π-stacking established by the twelve peptides have been reported in Table S3 in Supplementary materials. 

Finally, MM-GBSA calculations were performed and the results are reported in Table 5. Even for these peptides, the resulting ΔG_binding_ average values were compared to those retrieved from MD simulations of HOXA9 and HTL001 hexapeptides in complex with PBX1 and DNA. Only the sixth peptide showed a higher ΔG_binding_ average value, hence it was not considered for further studies.

**Figure 6:**
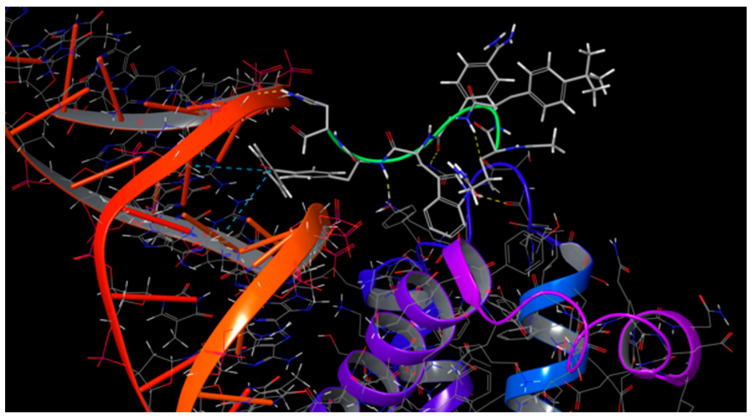
Binding mode of the first combinatorial peptide to PBX1 and DNA.

The analysis of the MD trajectories shed light on an interesting observed binding behaviour of the combinatorial peptides. Indeed, the tryptophan amino acid was kept stuck within the hydrophobic pocket of PBX1 during all the twelve MD simulations. Moreover, the selected non-standard amino acids in position X_1_ of the designed peptides presented hydrophobic side chains, as required from the consensus HOX hexapeptide [18]. These amino acids in position X_1_ showed also to stack their side chain into the DNA minor groove by establishing π-stacking contacts with the nitrogenous bases (Figure 6). Moreover, the interactions between DNA and combinatorial peptides were also explored and Table S4 depicts the interaction histograms for each complex DNA-PBX1-peptide in Supplementary materials. The analysis of the histogram plots highlighted three combinatorial peptides with the lowest ΔG_binding_ values (i.e., ΔG_first_peptide_ = –79.6771 kcal/mol, ΔG_fifth_peptide_ = –81.8766 kcal/mol and ΔG_ninth_peptide_ = –74.0909 kcal/mol, respectively) as the ones establishing π-stacking interaction with the DNA minor groove, especially with the deoxyguanosine 28 (DG28). Furthermore, hydrophobic interactions and H-bonds between peptides and DNA also seemed to be important for the ternary complex stabilisation. In this case, DG28 had again an important role for the stabilisation, being, together with deoxyadenosine 16 (DA16), the main residue involved in H-bonds interactions. Indeed, the sixth peptide, which reported a ΔG_binding_ value of –55.1927 kcal/mol, exhibited no π-stacking and H-bonds and very few hydrophobic contacts with DNA. These findings suggested that the contacts between combinatorial peptides and DNA minor groove might contribute to stabilising the complex DNA-PBX1-peptide. Table S5 in Supplementary materials illustrates the combinatorial peptide binding modes with PBX1 protein and DNA.

Finally, considering that both proteins HOX and PBX are involved in transcriptional events, to check the potential ability of the combinatorial peptides to permeate cell membranes [58], the polar surface area (PSA) and logP_o/w_ [59] were computed and Table 6 lists the values of the twelve designed peptides.

## 3. Methods

### 3.1. Preparation of HOXA9-PBX1-DNA Complex

The 3D trimeric complex of HOXA9-PBX1-DNA was downloaded from the Protein Data Bank [51] (PDB ID: 1PUF) and imported in Schrödinger suite (Schrödinger Inc., New York, NY, USA, software release v2018-4,) to optimise the structure by using the "Protein preparation" tool [60]. The bond orders for untemplated residues were assigned by using known HET groups based on their SMILES strings in Chemical Component Dictionary. Hydrogen atoms were added to the structure, eventual bonds to metals were broken, zero-order bonds between metals and nearby atoms were added and formal charges to metals and neighbouring atoms were corrected. Disulphide bonds between two sulphur atoms, if they were close to each other, were created and water molecules beyond 5.0 Å from any of the HET groups, including ions, were deleted. Then, protonation and metal charge states for the ligands, cofactors and metals were generated [61,62]. Finally, PROPKA [62] was run under pH 7.0 to optimise hydroxyl, Asn, Gln and His states using ProtAssign.

### 3.2. HOXA9 Hexapeptide Residues Scanning Using Non-standard “SwissSidechain” Amino Acids

The SwissSidechain database of non-natural amino acids was downloaded from the Swiss Institute of Bioinformatics website [53] as a .nsr file including parameter and topology data [54]. It was imported in Schrödinger tool “Manage non-standard amino acids” and joined with the non-natural residue library available in the Schrödinger suite, achieving overall 220 non-standard amino acids. The joined database was used to perform point mutations through the “Residue Scanning” tool of Schrödinger suite on HOXA9 residues (Ala196, Ala197, Asn198, Trp199, Leu200 and His201) by using PDB 1PUF [27]. 

The predicted changes in binding affinity and stability were calculated according to Equation (1) [56]. The resulting structures were refined by selecting side-chain prediction with backbone minimization.

(1) $$\Delta \Delta G_{Affinity}=\left(E_{A\Delta B}^{MUT}-E_{A}^{MUT}-E_{B}^{MUT}\right)-\left(E_{A\Delta B}^{WT}-E_{A}^{WT}-E_{B}^{WT}\right)$$

Where *E* is the calculated energy of each protein (*A* and *B*) or complex (*A*∙*B*) after refinement considering the mutant form (*MUT*) and the wild-type (*WT*) of the protein.

∆∆*G_stability_* values are calculated according to Equation (2). For the purpose of the model, ∆∆*G_stability_* was computed representing the unfolded ligand as a tripeptide, A-X-B, where X is the residue that is mutated, and A and B are its neighbours, capped with ACE and NMA. The assumption is that the remaining interactions in the unfolded state are negligible.

(2) $$\Delta \Delta G_{Stability}=\left(E_{L\left(u\right)}^{MUT}-E_{L\left(f\right)}^{MUT}\right)-\left(E_{L\left(u\right)}^{WT}-E_{L\left(f\right)}^{WT}\right)$$

Where *E*, in this case, is the calculated energy for the unfolded parent ligand (*L(u)*) and the folded parent ligand (*L(f)*) considering the mutant form (*MUT*) and the wild-type (*WT*) of the protein [56]. The calculations are done with Prime MM-GBSA [63,64], which employs an implicit (continuum) solvation model.

### 3.3. MD Simulations of PBX1-DNA in Complex with HOXA9 Protein, HOXA9 Hexapeptide, HTL001 Core Peptide, Point-mutated Peptides and Combinatorial Peptides

In this work overall, thirty-five MD simulations of 200 ns per each were performed using Desmond [65] and they were run by applying the following settings. The systems were created using the "System builder" tool of the Schrödinger suite. TIP3P [66] was selected as a solvent model and the orthorhombic shape box was chosen. The selected box size calculation method was buffer and the box side distances were set 10 Å. The force field OPLS3 [67] was applied and the system was neutralized by adding Na^+^ ions. The outputs were further processed by performing MD simulations of 200 ns. The ensemble class NPT was chosen to maintain the number of atoms, the pressure and the temperature constant for the entire trajectories. The thermostat method employed was the Nose--Hoover chain with a relaxation time of 1.0 ps and a temperature of 300 K. The barostat method applied was Martyna-Tobias-Klein with a relaxation time of 2.0 ps and an isotropic coupling style. The timestep for numerical integration was 2.0 fs for bonded interactions, 2.0 fs for nonbonded near (van der Waals and short-range electrostatic interactions), and 6.0 fs for nonbonded-far (long-range electrostatic interactions). For Coulombic interactions, a cut-off radius of 9.0 Å was tuned as a short-range method. Pressure and temperature were set at 1.01325 bar and 300 K, respectively. Finally, the systems were relaxed before starting simulations according to the following steps:

(1) Minimization with the solute restrained;
(2) Minimization without restraints;
(3) Simulation in the NVT ensemble using a Berendsen thermostat with a simulation time of 12 ps, a temperature of 10 K, a fast temperature relaxation constant, velocity resampling every 1 ps, and non-hydrogen solute atoms restrained;
(4) Simulation in the NPT ensemble using a Berendsen thermostat and a Berendsen barostat with a simulation time of 12 ps, a temperature of 10 K and a pressure of 1 atm, a fast temperature relaxation constant, a slow pressure relaxation constant, velocity resampling every 1 ps, and non-hydrogen solute atoms restrained;
(5) Simulation in the NPT ensemble using a Berendsen thermostat and a Berendsen barostat with a simulation time of 24 ps, a temperature of 300 K and a pressure of 1 atm, a fast temperature relaxation constant, a slow pressure relaxation constant, velocity resampling every 1 ps, and non-hydrogen solute atoms restrained;
(6) Simulation in the NPT ensemble using a Berendsen thermostat and a Berendsen barostat with a simulation time of 24 ps, a temperature of 300 K and a pressure of 1 atm, a fast temperature relaxation constant and a normal pressure relaxation constant.

### 3.4. MM-GBSA Calculations Performed on the Protein-peptide Complexes

The MD outputs of HOXA9 hexapeptide, HTL001 core peptide, point-mutated HOXA9 peptides and combinatorial peptides in complex with PBX1 protein and DNA were used to compute MM-GBSA calculations through the command line. For this purpose, the Python script “thermal_mmgbsa.py” was run.

Overall thirty-four MM-GBSA calculations were carried out using VSGB as a solvation model and OPLS3 FF was set for each MD trajectory. The Δ*G_binding_* values were computed for each trajectory frames according to Equation (3).

(3) $${\Delta G}_{binding}=E_{A\Delta B\;\left(minimized\right)}-E_{A\;\left(minimized\right)}-E_{B\;\left(minimized\right)}$$

Where *E* is the calculated energy of complex (*A*∙*B*) or each protein (*A* and *B*) after minimization [68]. Finally, the average of Δ*G_binding_* values of the entire trajectories was calculated and the results are above reported in Table 2, Table 3 and Table 4 of the “Results and discussion” section.

## 4. Conclusions

This work was based on the use of computational methods to design new non-standard peptides to inhibit HOX-PBX1 interaction. The above-described methods started from literature evidence [3,14,18,27,31,32] as an initial guide for the in silico design of new peptide inhibitors. In details, MD simulations of the newly designed peptides, in complex with PBX1 protein and DNA, reported promising results according to the binding mode, the predicted ΔG_binding_ average values and the physicochemical properties. From our extensive analysis, we got some important hints for the design of these new inhibitors. The first amino acid of the sequence should be hydrophobic and possibly aromatic to establish a π-stacking interaction with the DNA minor groove. The TRP in the fourth position seemed to be crucial for the stabilisation of the complex. From our computational analysis, the sixth amino acid (HIS) appeared not to be substitutable. 

The most promising peptides found out from this work will be soon synthesized, to test their efficacy in preventing HOX-PBX1 cooperative binding. In detail, biological assays will be conducted on ovarian, breast, and melanoma cancer cell lines, because of the overexpression of *HOX* genes.

## Figures and Tables

**Table 1:** The most frequent interactions and the related involved residues for HOXA9 hexapeptide 196-AANWLH-201 and PBX1 homeodomain proteins retrieved from MD frames clustering.

HOXA9 Residue	PBX1 Residue	Interaction Type
Trp199	Ser257	1 H-bond
Trp199	Leu256	1 H-bond
Trp199	Tyr291	π-stacking
Trp199	Tyr260	π-stacking
Leu200	Tyr260	1 H-bond
Ala197	Asn258	1 H-bond

**Table 2:** MM-GBSA calculation results of MD simulations performed on HOXA9 and HTL001 hexapeptides in complex with PBX1 protein and DNA.

Peptide Involved	HOXA9 Hexapeptide	HTL001 Hexapeptide
ΔG_binding_ average (kcal/mol)	−58.1922	−53.6882
ΔG_binding_ Std. Dev.	8.99	8.53
ΔG_binding_ range (kcal/mol)	−84.6286 to −34.1107	−78.0904 to −28.9169

**Table 3:** The best four non-standard amino acids selected through residue scanning calculations according to ΔΔG_affinity_ and ΔΔG_stability_.

Corresponding HOXA9 aa	Non-natural Amino Acid	Non-natural Amino Acid Structure	ΔΔG_affinity_	ΔΔG_stability_
**ALA196**	CIR	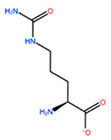	−45.128 kcal/mol	−0.816 kcal/mol
	ALC	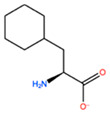	−18.704 kcal/mol	−4.095 kcal/mol
	MTR	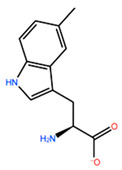	−17.088 kcal/mol	−3.208 kcal/mol
	CTE	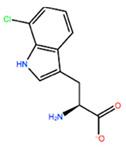	−15.008 kcal/mol	−5.778 kcal/mol
**ALA197**	BIF	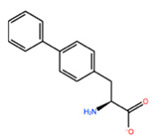	−12.892 kcal/mol	−3.615 kcal/mol
	TBP	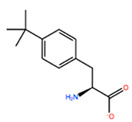	−8.133 kcal/mol	−3.556 kcal/mol
	HRG	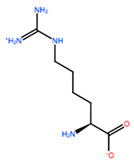	−6.681 kcal/mol	−9.341 kcal/mol
	CIR	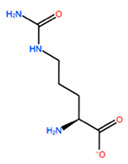	−5.075 kcal/mol	−2.606 kcal/mol
**ASN198**	MOT	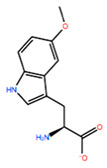	−7.505 kcal/mol	−13.303 kcal/mol
	0BN	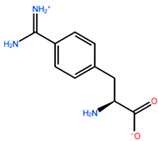	−6.051 kcal/mol	−10.151 kcal/mol
	KYN	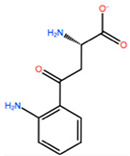	−6.041 kcal/mol	−5.511 kcal/mol
	GBU	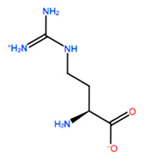	−5.867 kcal/mol	−0.688 kcal/mol
**LEU200**	PBF	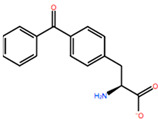	−51.368 kcal/mol	−3.302 kcal/mol
	CP3	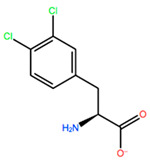	−11.929 kcal/mol	−1.106 kcal/mol
	QU4	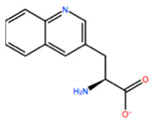	−11.415 kcal/mol	−4.372 kcal/mol
	ANT	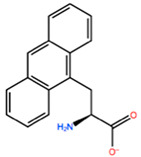	−11.134 kcal/mol	−1.353 kcal/mol
**HIS201**	ILX	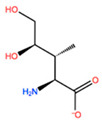	−11.562 kcal/mol	−1.371 kcal/mol
	HIL	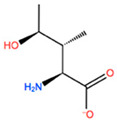	−10.330 kcal/mol	−2.198 kcal/mol
	DPP	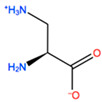	−4.195 kcal/mol	−1.029 kcal/mol
	HRG	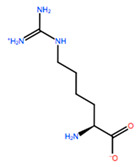	−4.018 kcal/mol	−5.686 kcal/mol

**Table 4:** Data results from MM-GBSA calculations of MD trajectories performed on point mutated HOXA9 peptides in complex with PBX1 and DNA

	**X_1_ = ALA196 Mutation**		**X_2_ = ALA197 Mutation**		**X_3_ = ASN198 Mutation**	
ΔG_binding_ average (kcal/mol)	**CIR**	−68.1064	**BIF**	−52.5114	**MOT**	−51.3925
ΔG_binding_ Std. Dev.		9.19		8.23		9.77
ΔG_binding_ range (kcal/mol)		−91.4424 to −33.6024		−77.3444 to −25.9087		−79.7187 to −21.3466
ΔG_binding_ average (kcal/mol)	**MTR**	−58.3419	**TBP**	−59.0603	**0BN**	−59.1051
ΔG_binding_ Std. Dev.		7.37		9.34		8.71
ΔG_binding_ range (kcal/mol)		−85.6739 to −35.5824		−80.7314 to −31.1314		−82.4310 to −27.3523
ΔG_binding_ average (kcal/mol)	**ALC**	−59.6952	**HRG**	−54.3707	**KYN**	−56.4406
ΔG_binding_ Std. Dev.		7.39		9.34		7.35
ΔG_binding_ range (kcal/mol)		−78.3738 to −26.9511		−85.1600 to −24.8071		−75.4170 to −28.6690
ΔG_binding_ average (kcal/mol)	**CTE**	−56.3011	**CIR**	−57.6592	**GBU**	−55.1339
ΔG_binding_ Std. Dev.		7.55		8.88		7.97
ΔG_binding_ range (kcal/mol)		−80.4156 to −29.7472		−81.7311 to −26.2628		−77.5916 to −30.5304
	**X**_**4**_ **= LEU200 Mutation**		X_**5**_ **= HIS201 Mutation**			
ΔG_binding_ average (kcal/mol)	**PBF**	−68.1857	**ILX**	−48.9087		
ΔG_binding_ Std. Dev.		8.44		8.81		
ΔG_binding_ range (kcal/mol)		−95.1687 to −39.7321		−74.3783 to −20.2857		
ΔG_binding_ average (kcal/mol)	**CP3**	−64.6802	**HIL**	−50.3148		
ΔG_binding_ Std. Dev.		9.49		9.03		
ΔG_binding_ range (kcal/mol)		−89.3192 to−34.0239		−79.2197 to −22.2265		
ΔG_binding_ average (kcal/mol)	**QU4**	−61.8016	**DPP**	−55.6169		
ΔG_binding_ Std. Dev.		10.53		11.48		
ΔG_binding_ range (kcal/mol)		−88.4144 to −33.9895		−91.1993 to −22.9813		
ΔG_binding_ average (kcal/mol)	**ANT**	−63.3043	**HRG**	−57.0861		
ΔG_binding_ Std. Dev.		8.50		9.46		
ΔG_binding_ range (kcal/mol)		−87.5841 to −35.9672		−86.8681 to −27.0434		

**Table 5:** MM-GBSA calculation results of MD simulations performed on the combinatorial peptides in complex with PBX1 protein and DNA.

**PEPTIDE INVOLVED**	**First Peptide**	**Second Peptide**	**Third Peptide**
ΔG_binding_ average	−79.6771 kcal/mol	−61.8602 kcal/mol	−68.0795 kcal/mol
ΔG_binding_ Std. Dev.	10.18	12.72	10.63
ΔG_binding_ range	−104.585 to −38.2615 kcal/mol	−99.0013 to −30.7190 kcal/mol	−98.3690 to −29.5313 kcal/mol
**PEPTIDE INVOLVED**	**Fourth Peptide**	**Fifth Peptide**	**Sixth Peptide**
ΔG_binding_ average	−64.6664 kcal/mol	−81.8766 kcal/mol	−55.1927 kcal/mol
ΔG_binding_ Std. Dev.	7.53	7.44	10.09
ΔG_binding_ range	−87.5689 to −30.1013 kcal/mol	−101.5164 to −45.3623 kcal/mol	−85.5158 to −22.5652 kcal/mol
**PEPTIDE INVOLVED**	**Seventh Peptide**	**Eighth Peptide**	**Ninth Peptide**
ΔG_binding_ average	−62.8885 kcal/mol	−71.9163 kcal/mol	−74.0909 kcal/mol
ΔG_binding_ Std. Dev.	9.19	9.19	11.42
ΔG_binding_ range	−89.1247 to −19.4438 kcal/mol	−101.6790 to −44.4808 kcal/mol	−105.5444 to −32.2303 kcal/mol
**PEPTIDE INVOLVED**	**Tenth Peptide**	**Eleventh Peptide**	**Twelfth Peptide**
ΔG_binding_ average	−60.2167 kcal/mol	−65.0198 kcal/mol	−68.3222 kcal/mol
ΔG_binding_ Std. Dev.	9.56	8.24	8.13
ΔG_binding_ range	−89.0633 to −28.6783 kcal/mol	−89.3709 to −36.2812 kcal/mol	−95.3406 to −42.1593 kcal/mol

**Table 6:** PSA and logP_o/w_ values of the twelve combinatorial peptides.

PEPTIDES		PSA	logP_o/w_
1	CIR-TBP-0BN-TRP-PBF-HIS	302.15	3.2
2	CIR-TBP-0BN-TRP-CP3-HIS	310.38	2.6
3	CIR-TBP-0BN-TRP-QU4-HIS	322.80	2.0
4	CIR-TBP-0BN-TRP-ANT-HIS	307.47	3.3
5	ALC-TBP-0BN-TRP-PBF-HIS	300.04	6.1
6	ALC-TBP-0BN-TRP-CP3-HIS	284.52	6.0
7	ALC-TBP-0BN-TRP-QU4-HIS	297.08	5.4
8	ALC-TBP-0BN-TRP-ANT-HIS	250.48	5.9
9	MTR-TBP-0BN-TRP-PBF-HIS	276.41	4.3
10	MTR-TBP-0BN-TRP-CP3-HIS	268.43	5.4
11	MTR-TBP-0BN-TRP-QU4-HIS	280.80	4.8
12	MTR-TBP-0BN-TRP-ANT-HIS	260.87	6.0

## Supplementary Materials

The following are available online at https://www.mdpi.com/article/10.3390/ijms22115670/s1.
