# Supplementary material for: A Computer-Based Methodology to Design Non-Standard Peptides Potentially Able to Prevent HOX-PBX1-Associated Cancer Diseases
Source: Int J Mol Sci. 2021 May 26;22(11):5670. doi: 10.3390/ijms22115670 (PMC8198631; doi:10.3390/ijms22115670)
Supplement: Supplementary file 1 [file ijms-22-05670-s001.zip › ijms-1209267-supplementary.pdf]

Supplementary materials

Title: A computer-based methodology to design non-standard peptides potentially able to prevent HOX-PBX1-associated cancer diseases

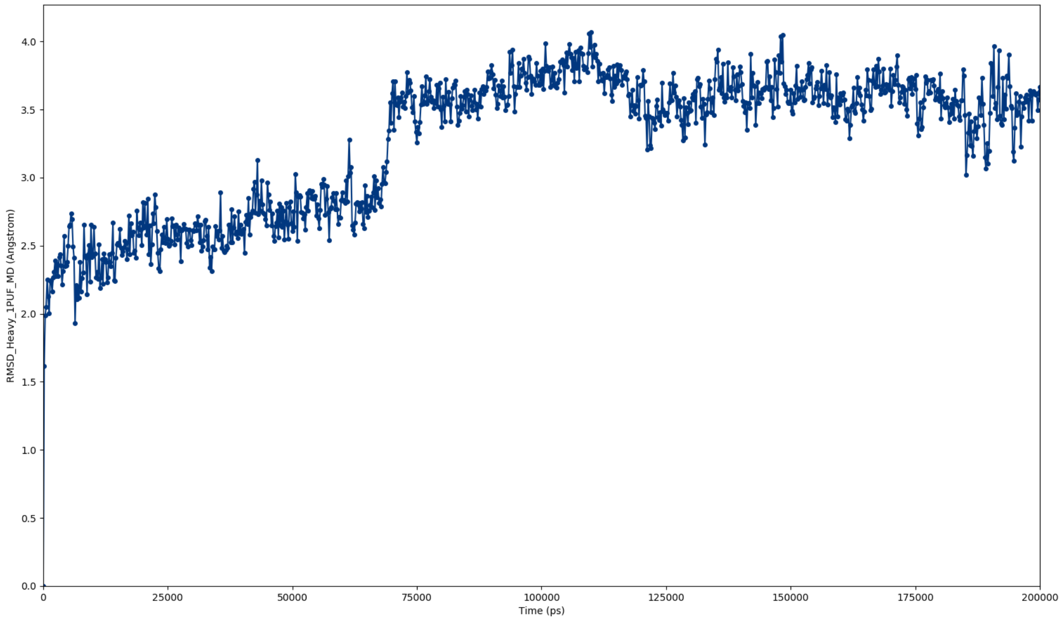

Figure S1. RMSD plot of MD simulation of 200 ns performed on HOXA9-PBX1-DNA complex (PDB ID:1PUF)

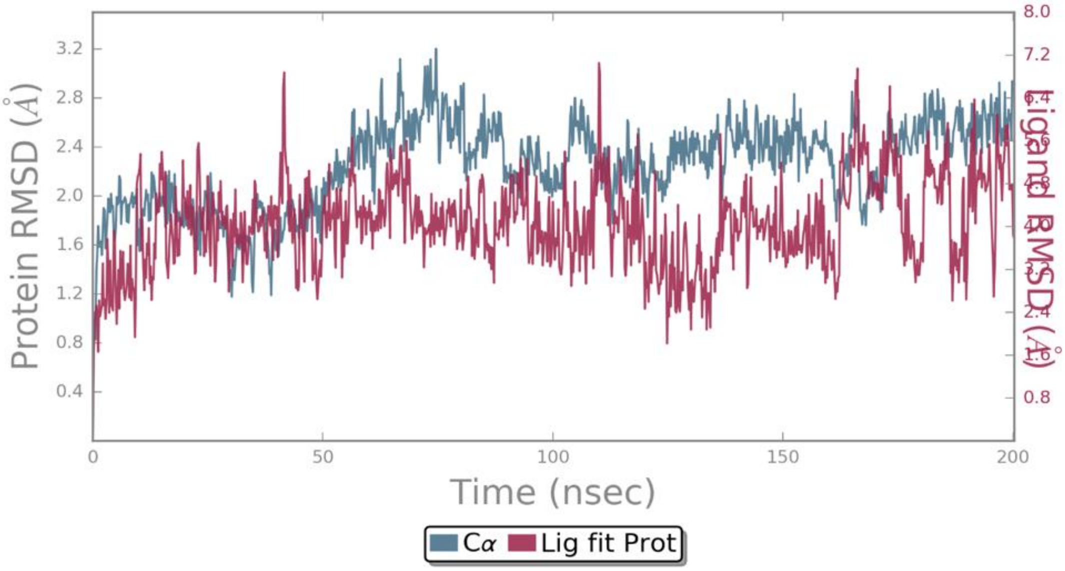

Figure S2. RMSD plot of PBX1 protein and HOXA9 hexapeptide 196-AALWNH-201 during the MD trajectory

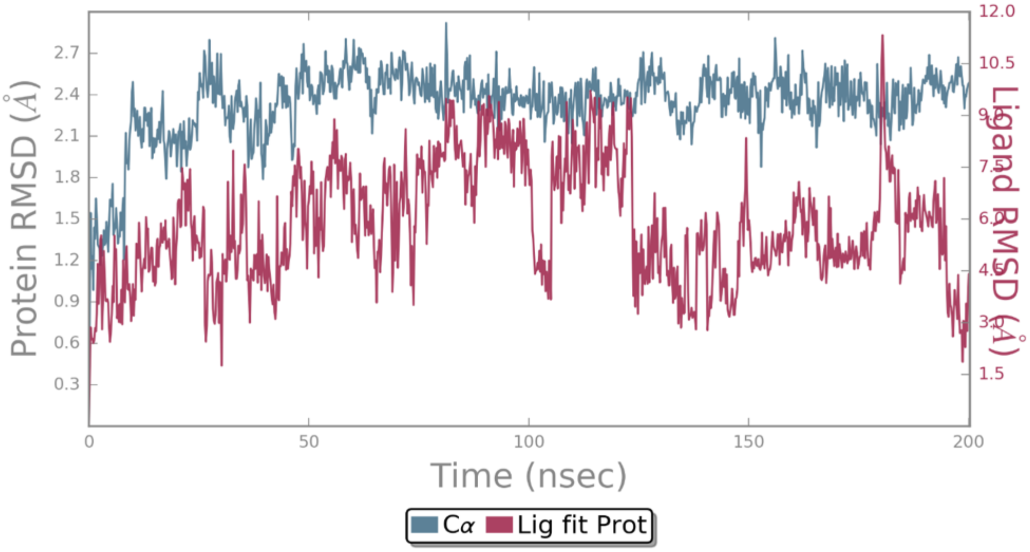

Figure S3. RMSD plot of PBX1 protein and HTL001 hexapeptide WYKWMK during the MD trajectory

Table S1. RMSD plots of HOXA9-based combinatorial peptides in complex with PBX1 protein and DNA

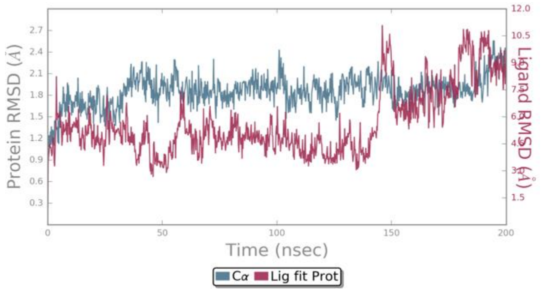 **First Peptide**	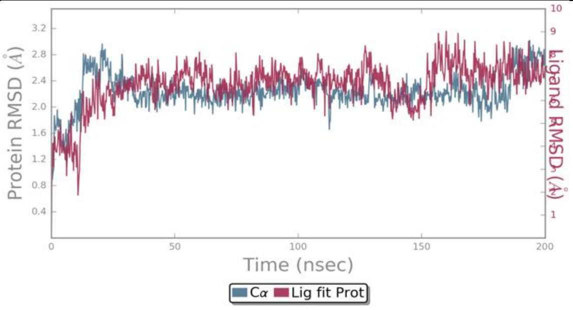 **Second Peptide**
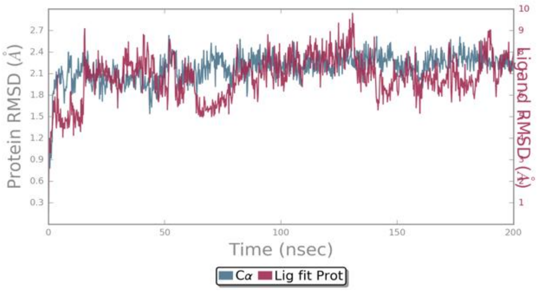 **Third Peptide**	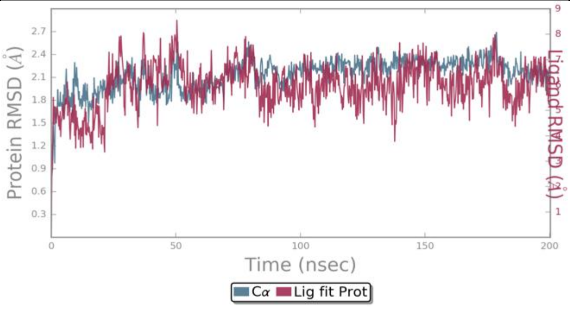 **Fourth Peptide**
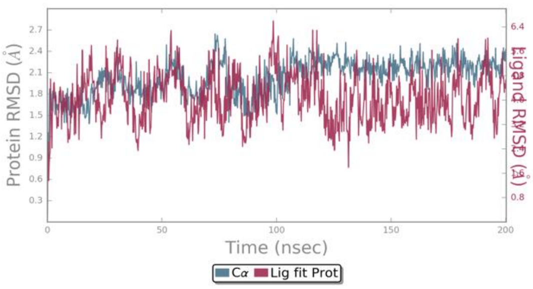 **Fifth Peptide**	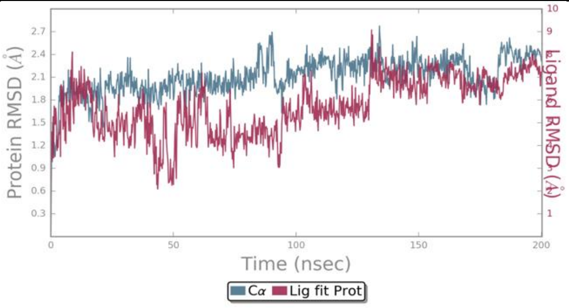 **Sixth Peptide**

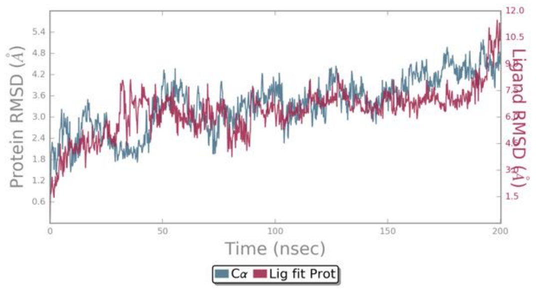

Seventh Peptide

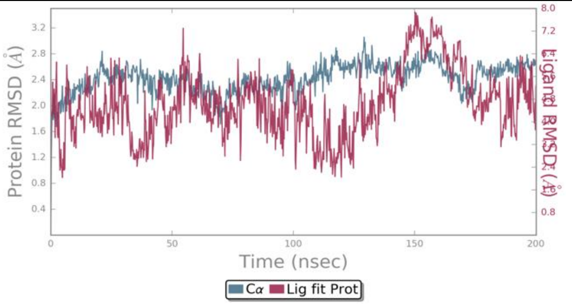

Eighth Peptide

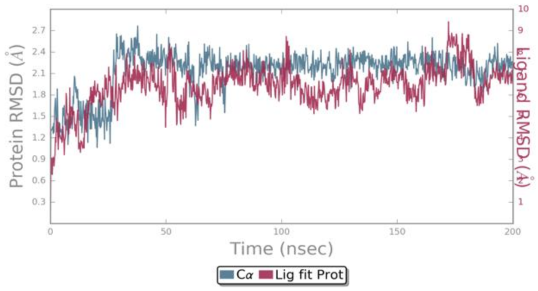

Ninth Peptide

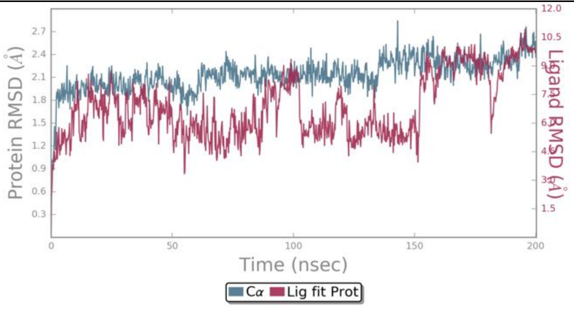

Tenth Peptide

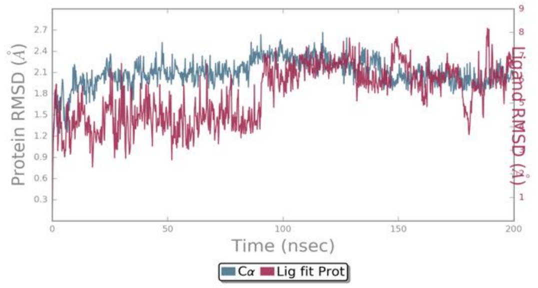

Eleventh Peptide

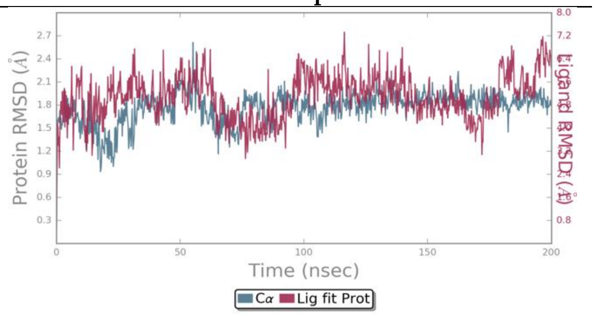

Twelfth Peptide

Table S2. The bar charts of protein-ligand interactions for the twelve HOXA9-based combinatorial peptides (on the left column); the plots illustrating the frequency of interaction occurrences between the combinatorial peptides and PBX1 protein (on the right column)

First Peptide

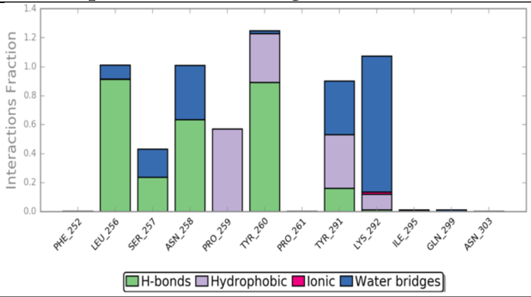

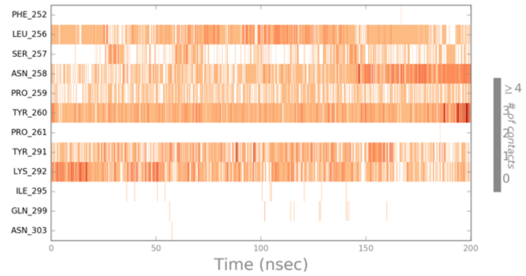

Second Peptide

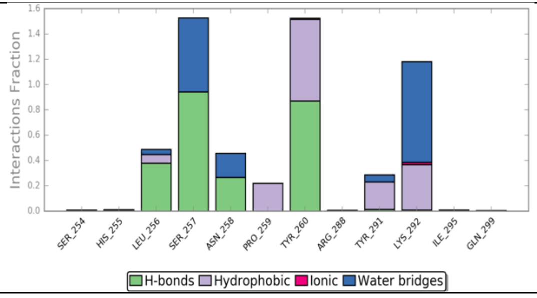

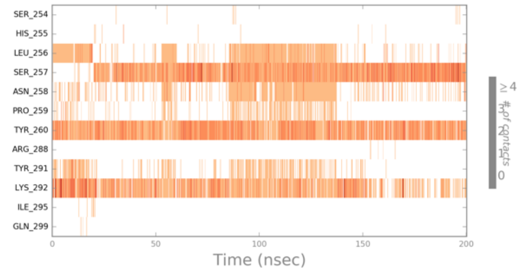

Third Peptide

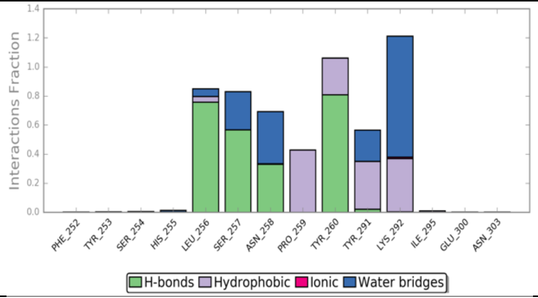

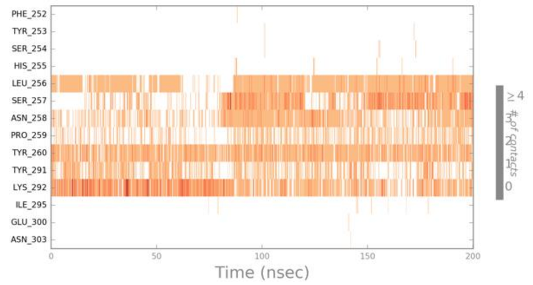

Fourth Peptide

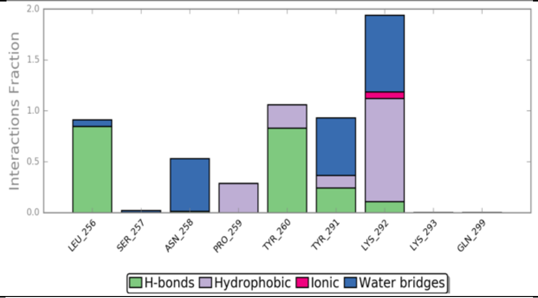

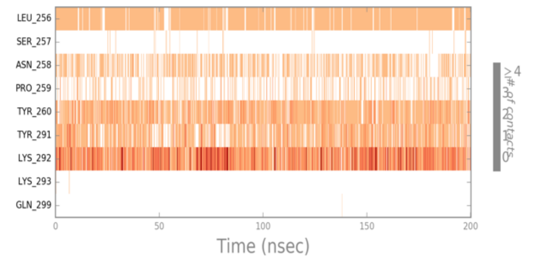

Fifth Peptide

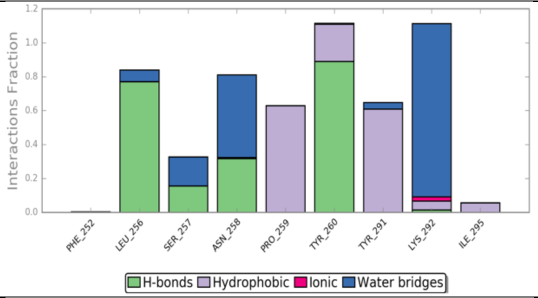

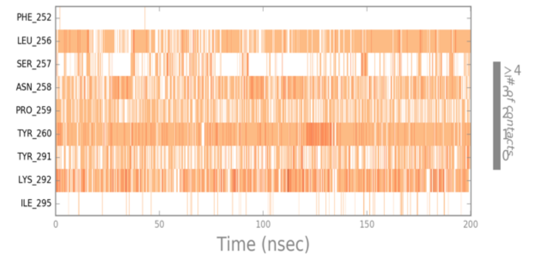

Sixth Peptide

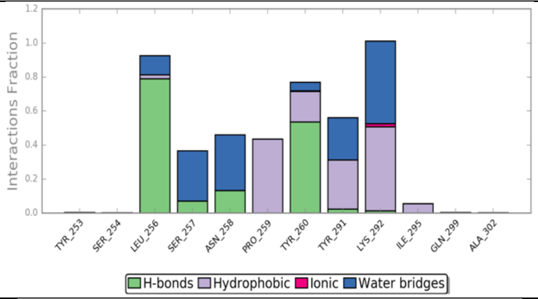

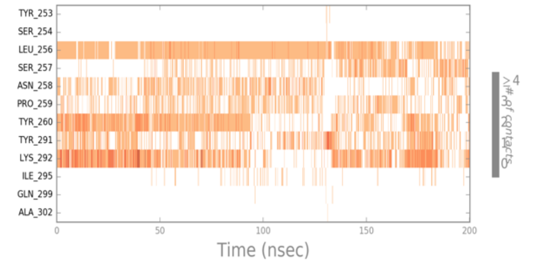

Seventh Peptide

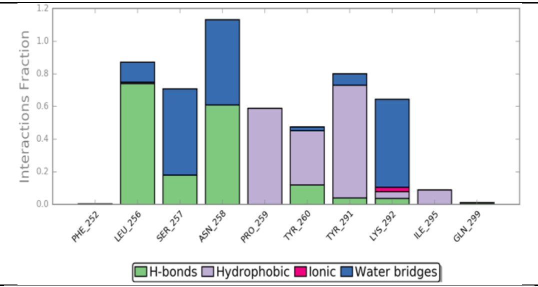

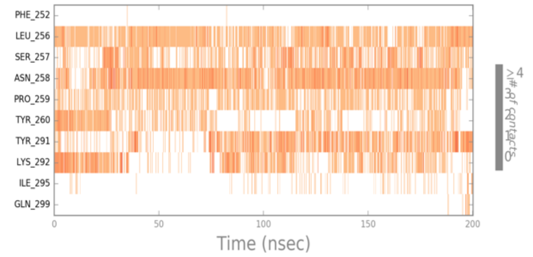

Eighth Peptide

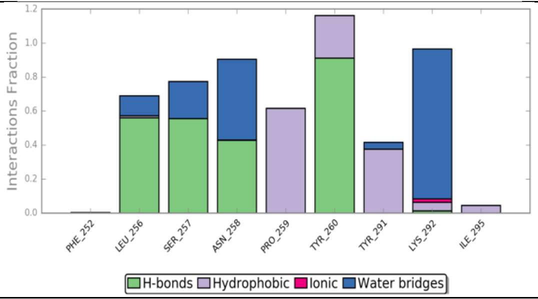

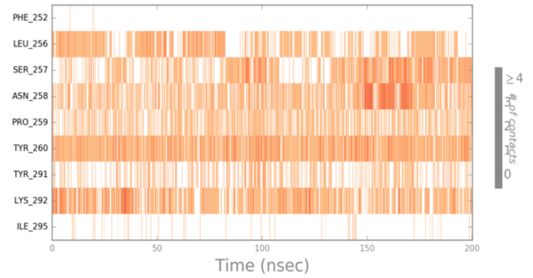

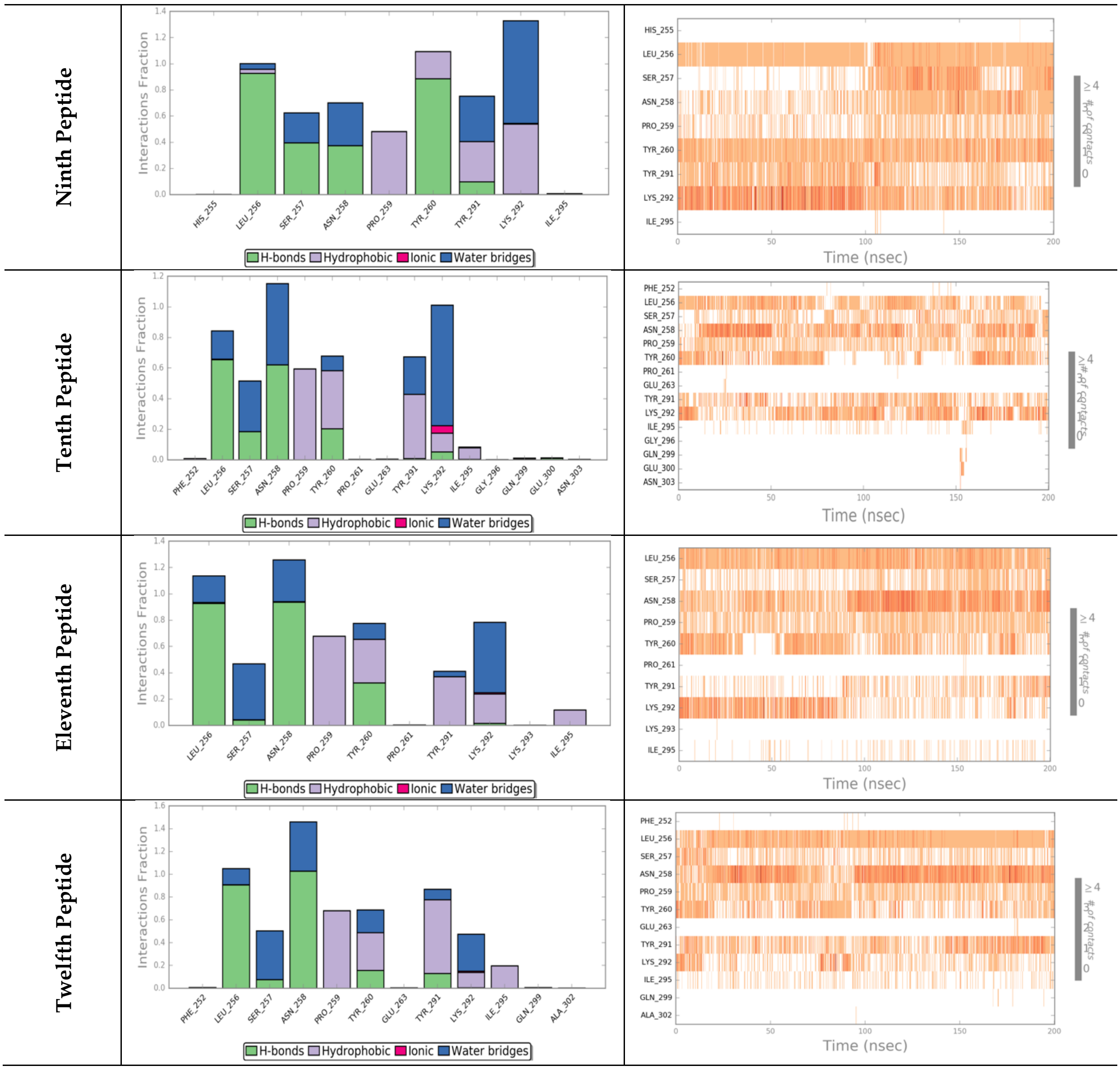

Table S3. Statistics data of H-bonds and π-stacking established by the twelve combinatorial peptides with PBX1 protein.

	H-BONDS		
	**Residue**	**Occurrence Mean**	**Occurrence Mean Std. deviation**
First Peptide	LEU_256	0.321	0.467
	TYR_260	0.313	0.464
	LYS_292	0.004	0.062
	ASN_258	0.223	0.416
	TYR_291	0.056	0.231
	SER_257	0.083	0.276
Second Peptide	TYR_260	0.351	0.477
	LYS_292	0.003	0.057
	LEU_256	0.152	0.359
	TYR_291	0.005	0.072
	SER_257	0.380	0.485

	HIS_255	0.002	0.040
	ASN_258	0.107	0.309
**Third Peptide**	SER_257	0.228	0.420
	TYR_260	0.325	0.469
	LYS_292	0.001	0.035
	LEU_256	0.305	0.460
	ASN_258	0.133	0.340
	TYR_291	0.008	0.089
**Fourth Peptide**	TYR_260	0.407	0.491
	LYS_292	0.052	0.223
	LEU_256	0.415	0.493
	TYR_291	0.119	0.324
	ASN_258	0.006	0.079
	SER_257	0.001	0.022
**Fifth Peptide**	SER_257	0.072	0.259
	LEU_256	0.359	0.480
	TYR_260	0.414	0.493
	LYS_292	0.007	0.080
	ASN_258	0.148	0.355
**Sixth Peptide**	TYR_260	0.343	0.475
	LYS_292	0.008	0.087
	LEU_256	0.506	0.500
	TYR_291	0.014	0.118
	ASN_258	0.085	0.278
	SER_257	0.045	0.207
**Seventh Peptide**	LEU_256	0.428	0.495
	TYR_260	0.069	0.253
	LYS_292	0.021	0.145
	ASN_258	0.352	0.478
	SER_257	0.104	0.305
	TYR_291	0.023	0.148
	GLN_299	0.004	0.063
**Eighth Peptide**	TYR_260	0.370	0.483
	LYS_292	0.005	0.072
	LEU_256	0.227	0.419
	ASN_258	0.173	0.378
	SER_257	0.225	0.418
**Ninth Peptide**	TYR_260	0.331	0.471
	LYS_292	0.001	0.033
	LEU_256	0.346	0.476
	TYR_291	0.036	0.187
	ASN_258	0.139	0.346
	SER_257	0.147	0.354
**Tenth Peptide**	TYR_260	0.116	0.321
	LYS_292	0.030	0.170
	LEU_256	0.376	0.485
	TYR_291	0.005	0.068
	SER_257	0.106	0.308
	ASN_258	0.357	0.479
	GLN_299	0.003	0.059
	GLU_300	0.005	0.072
	ILE_295	0.001	0.034

Eleventh Peptide	TYR_260	0.144	0.351
	LYS_292	0.007	0.081
	LEU_256	0.414	0.493
	ASN_258	0.418	0.493
	SER_257	0.018	0.132
Twelfth Peptide	LEU_256	0.395	0.489
	TYR_260	0.068	0.252
	ASN_258	0.447	0.497
	SER_257	0.033	0.177
	TYR_291	0.056	0.229
	LYS_292	0.002	0.042
	GLN_299	0.001	0.021

	π-STACKING					
	**Residue**	**π-π Type**[a]	**Occurrence Mean**	**Occurrence Mean Std. deviation**	**Distance Mean (Å)**	**Distance Mean Std. Deviation**
First Peptide	TYR_260	e2f	0.915	0.282	5.410	0.081
	TYR_291	e2f	0.043	0.204	5.352	0.105
	TYR_291	f2f	0.043	0.204	4.233	0.206
Second Peptide	TYR_260	e2f	0.980	0.141	5.147	0.193
	TYR_260	f2f	0.017	0.129	3.935	0.335
	TYR_291	e2f	0.003	0.058	5.138	-
Third Peptide	TYR_260	e2f	0.600	0.503	5.394	0.105
	TYR_260	f2f	0.050	0.224	3.806	-
	TYR_291	e2f	0.300	0.470	5.305	0.212
	TYR_291	f2f	0.050	0.224	4.232	-
Fourth Peptide	TYR_260	e2f	0.937	0.245	5.091	0.250
	TYR_291	e2f	0.063	0.245	5.286	0.217
Fifth Peptide	TYR_260	e2f	0.716	0.453	5.190	0.278
	TYR_260	f2f	0.049	0.217	3.984	0.235
	TYR_291	e2f	0.225	0.420	5.239	0.169
	TYR_291	f2f	0.010	0.099	4.272	-
Sixth Peptide	TYR_260	e2f	0.544	0.502	5.202	0.221
	TYR_260	f2f	0.103	0.306	3.967	0.143
	TYR_291	e2f	0.338	0.477	5.254	0.187
	TYR_291	f2f	0.015	0.121	4.314	-
Seventh Peptide	TYR_260	e2f	0.691	0.463	5.148	0.201
	TYR_260	f2f	0.029	0.168	4.071	0.193
	TYR_291	e2f	0.276	0.448	5.248	0.191
	TYR_291	f2f	0.004	0.064	4.264	-
Eighth Peptide	TYR_260	e2f	0.929	0.258	5.197	0.270
	TYR_291	e2f	0.071	0.258	5.279	0.168
Ninth Peptide	TYR_260	e2f	0.881	0.328	5.397	0.110
	TYR_260	f2f	0.024	0.154	3.698	-
	TYR_291	e2f	0.071	0.261	5.048	0.106
	TYR_291	f2f	0.024	0.154	4.286	-
Tenth Peptide	TYR_260	e2f	0.295	0.457	5.118	0.283
	TYR_260	f2f	0.456	0.499	3.905	0.236
	TYR_291	e2f	0.244	0.431	5.225	0.269
	TYR_291	f2f	0.005	0.068	3.900	-
Eleve nth Pepti	TYR_260	e2f	0.590	0.494	5.079	0.305
	TYR_260	f2f	0.230	0.422	4.076	0.200

	TYR_291	e2f	0.180	0.385	5.328	0.131
**Twelfth Peptide**	TYR_260	e2f	0.846	0.363	5.157	0.223
	TYR_291	e2f	0.140	0.348	5.294	0.184
	TYR_291	f2f	0.015	0.121	4.373	0.033

[a] "e2f" stands for edge-to-face and "f2f" for face-to-face.

Table S4. DNA-combinatorial peptide interaction histograms for the MD simulations of 200 ns

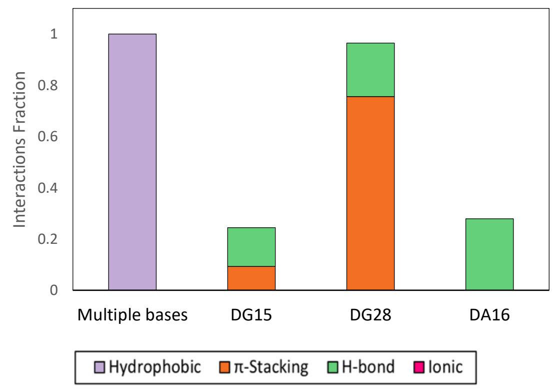

First Peptide

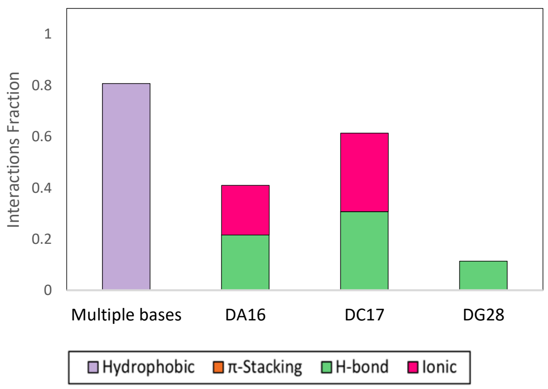

Second Peptide

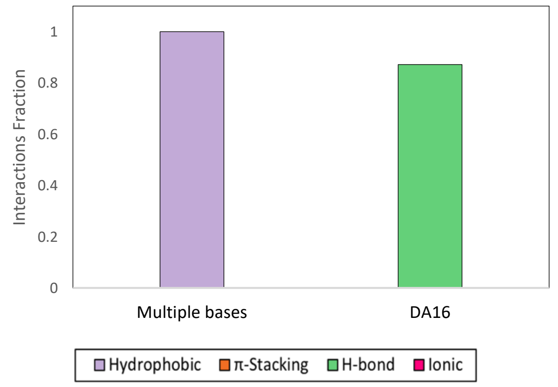

Third Peptide

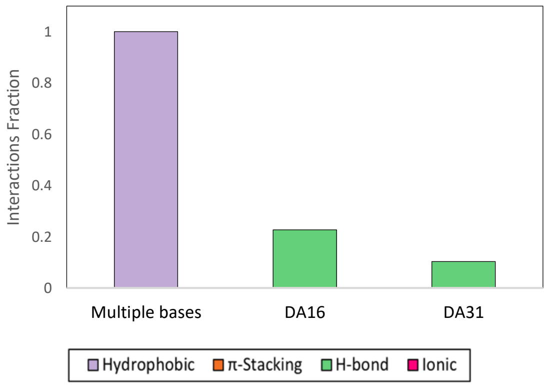

Fourth Peptide

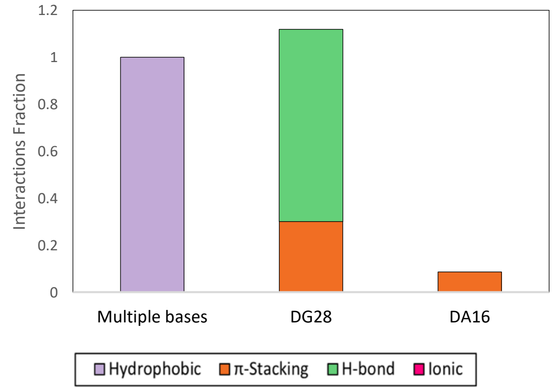

Fifth Peptide

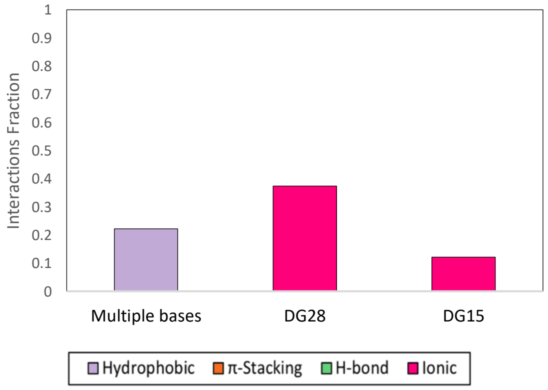

Sixth Peptide

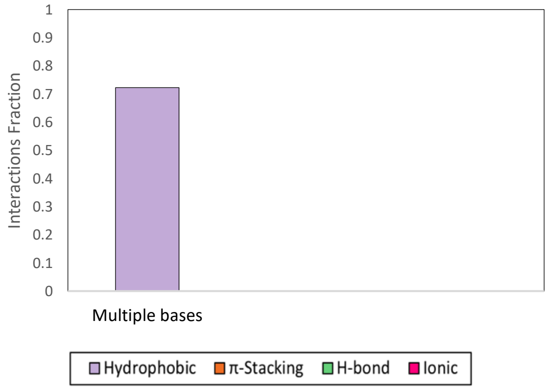

Seventh Peptide

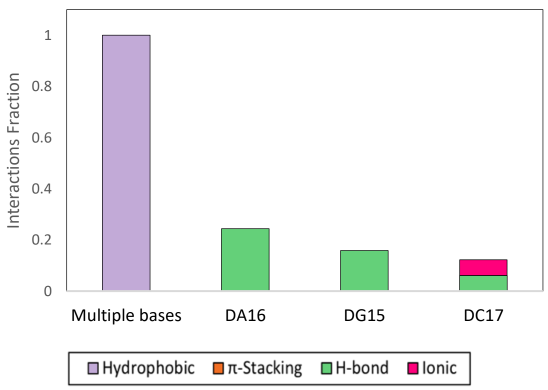

Eighth Peptide

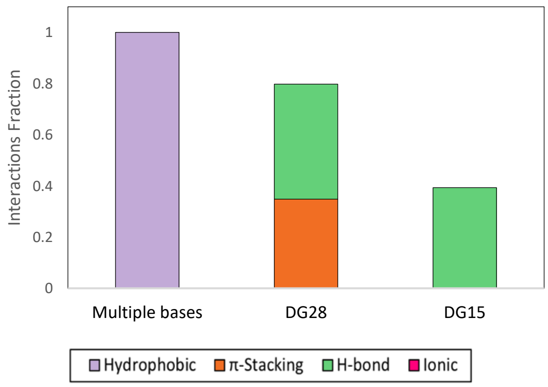

Ninth Peptide

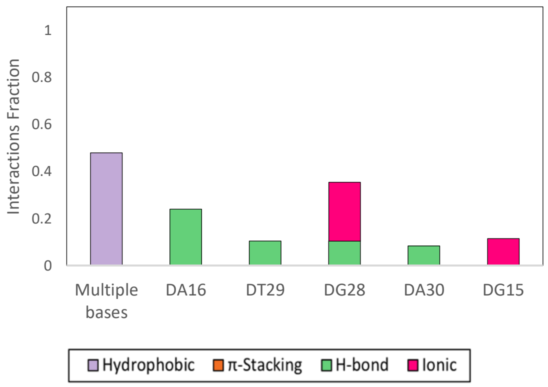

Tenth Peptide

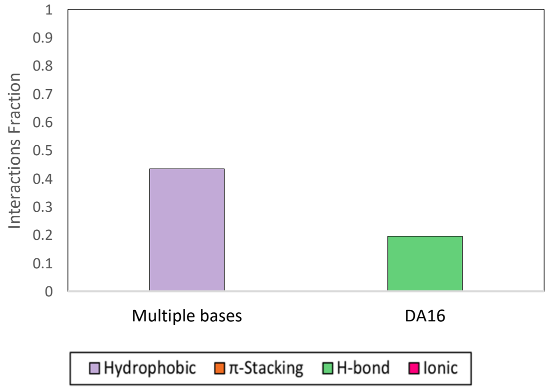

Eleventh Peptide

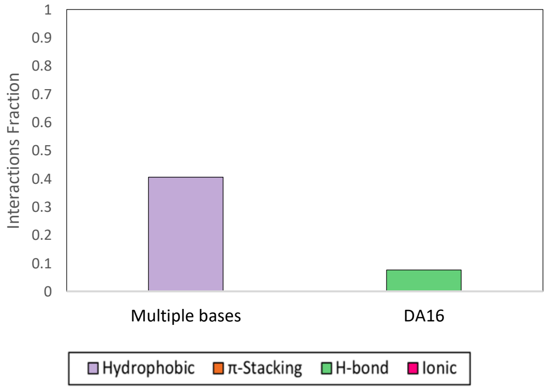

Twelfth Peptide

Table S5. Binding mode of the twelve combinatorial peptides in complex with PBX1 protein and DNA during MD simulations of 200 ns. Yellow-dotted lines stand for hydrogen bonds and blue-dotted lines are π-stacking

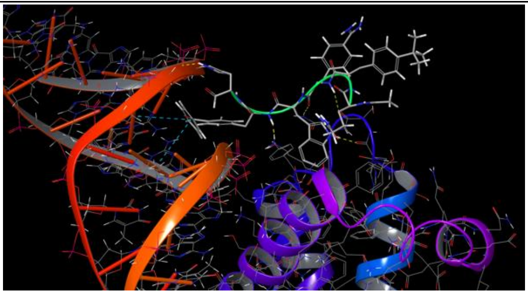

First Peptide

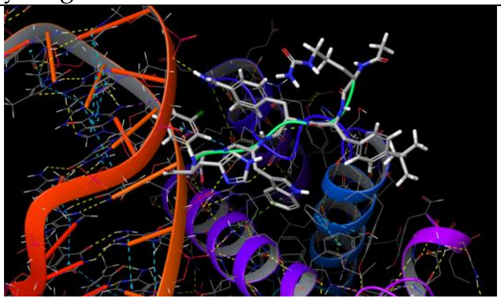

Second Peptide

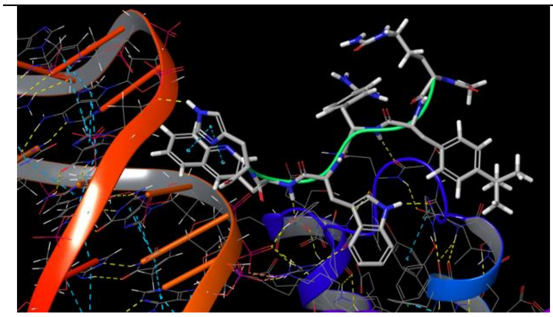

Third Peptide

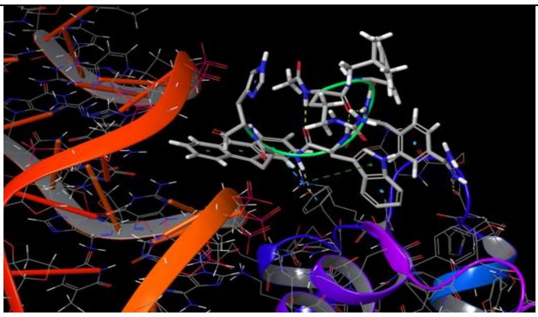

Fourth Peptide

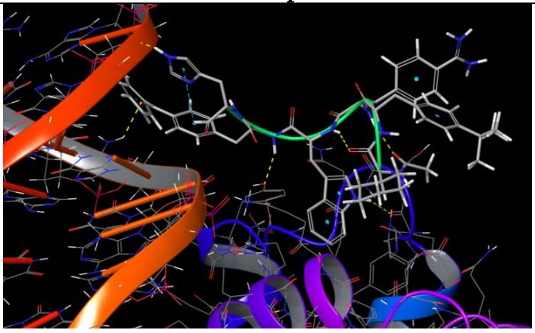

Fifth Peptide

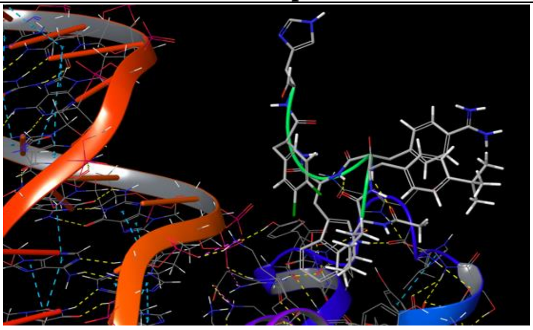

Sixth Peptide

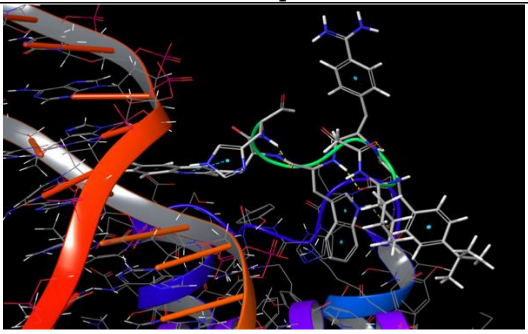

Seventh Peptide

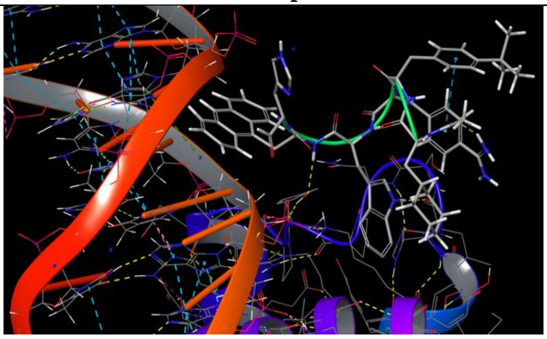

Eighth Peptide

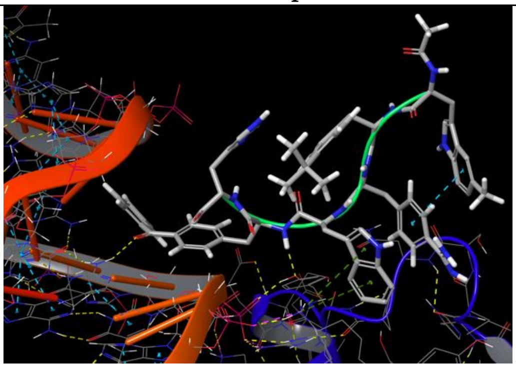

Ninth Peptide

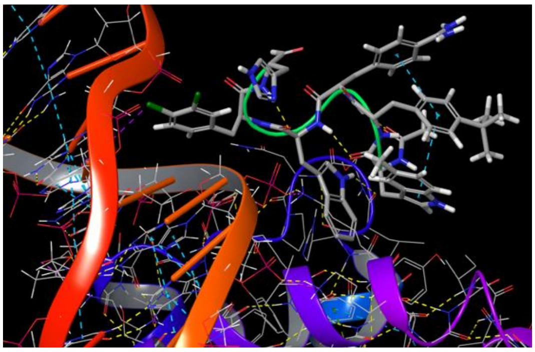

Tenth Peptide

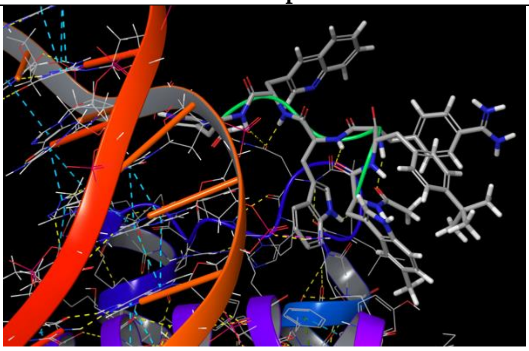

Eleventh Peptide

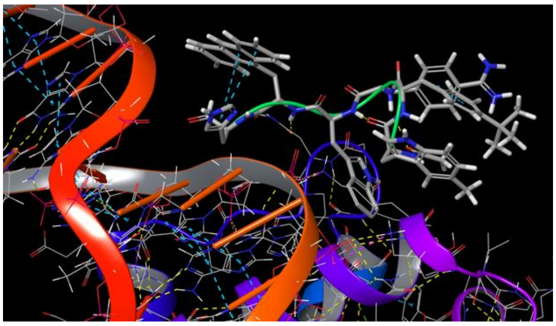

Twelfth Peptide
